# Yellow Fever Vaccine Booster Doses: Recommendations of the Advisory Committee on Immunization Practices, 2015

**Published:** 2015-06-19

**Authors:** J. Erin Staples, Joseph A. Bocchini, Lorry Rubin, Marc Fischer

**Affiliations:** 1 National Center for Emerging and Zoonotic Diseases, CDC; 2 Advisory Committee on Immunization Practices; 3 Louisiana State University Health Sciences Center, Shreveport, Louisiana; 4 North Shore-Long Island Jewish Health System, New Hyde Park, New York

On February 26, 2015, the Advisory Committee on Immunization Practices (ACIP) voted that a single primary dose of yellow fever vaccine provides long-lasting protection and is adequate for most travelers (1). ACIP also approved recommendations for at-risk laboratory personnel and certain travelers to receive additional doses of yellow fever vaccine (Box). The ACIP Japanese Encephalitis and Yellow Fever Vaccines Workgroup evaluated published and unpublished data on yellow fever vaccine immunogenicity and safety. The evidence for benefits and risks associated with yellow fever vaccine booster doses was evaluated using the Grading of Recommendations, Assessment, Development, and Evaluation (GRADE) framework (2,3). This report summarizes the evidence considered by ACIP and provides the updated recommendations for yellow fever vaccine booster doses.

## Yellow Fever Epidemiology and Risk for Disease in Travelers

Yellow fever is a mosquito-borne viral disease that is endemic to sub-Saharan Africa and tropical South America. Worldwide, yellow fever virus causes an estimated 200,000 cases of clinical disease and 30,000 deaths annually (4). Clinical disease ranges from a mild, nonspecific febrile illness to severe disease with jaundice and hemorrhage. The case-fatality ratio for severe yellow fever is 20%–50% (5). Because no specific treatment exists, prevention through vaccination is critical to reduce morbidity and mortality from yellow fever virus infection.

The risk of a traveler acquiring yellow fever varies based on season, location, activities, and duration of their travel. For a 2-week stay, the estimated risk for illness attributed to yellow fever for an unvaccinated traveler to West Africa is 50 cases per 100,000 population; for South America, the risk for illness is five cases per 100,000 population (6).

## Yellow Fever Vaccine Recommendations and International Health Regulations Requirements

Yellow fever vaccine is recommended for persons aged ≥9 months who are traveling to or living in areas with risk for yellow fever virus transmission (7). International Health Regulations allow countries to require proof of yellow fever vaccination from travelers entering their country (8). These requirements are intended to minimize the potential importation and spread of yellow fever virus. Currently, International Health Regulations specify that a dose of yellow fever vaccine is valid for 10 years. Therefore, at present, travelers to countries with a yellow fever vaccination entry requirement must have received a dose of yellow fever vaccine within the past 10 years.

### Recent changes to yellow fever vaccine recommendations

In April 2013, the World Health Organization Strategic Advisory Group of Experts on Immunization concluded that a single primary dose of yellow fever vaccine is sufficient to confer sustained immunity and lifelong protection against yellow fever disease, and that a booster dose is not needed (9). This conclusion was based on a systematic review of published studies on the duration of immunity after a single dose of yellow fever vaccine, and on data that suggest vaccine failures are extremely rare and do not increase in frequency with time since vaccination (10). The advisory group noted that future studies and surveillance data should be used to identify specific risk groups, such as persons infected with human immunodeficiency virus (HIV) or infants, who might benefit from a booster dose. In May 2014, the World Health Assembly adopted the recommendation to remove the 10-year booster dose requirement from the International Health Regulations by June 2016 (11).

## Yellow Fever Vaccine Long-term Immunogenicity Data

No data are available on vaccine efficacy or protective antibody titers (i.e., seroprotection) related to long-term immunogenicity after yellow fever vaccination. Benefits considered critical in assessing the need for booster doses of yellow fever vaccine for U.S. travelers or laboratory workers included vaccine effectiveness (i.e., a lack of vaccine failures) and evidence of seropositivity (i.e., yellow fever virus–specific antibodies detected in a blood sample) (3).

### Vaccine effectiveness

A total of 23 vaccine failures were identified after the administration of >540 million doses of yellow fever vaccine (3). Of the 23 cases, five occurred <10 days after vaccination and were excluded because most persons are not expected to develop protective titers in that timeframe (5). Of the remaining 18 cases, 16 (89%) occurred in persons who reported receiving a dose of the vaccine within the previous 10 years (3). One vaccine failure occurred at 20 years and one at 27 years post-vaccination.

### Seropositivity

Thirteen observational studies provided immunogenicity data on 1,137 persons vaccinated ≥10 years previously (3). Using a random effects model, the estimated seropositivity rate for persons vaccinated ≥10 years previously was 92% (95% confidence interval [CI] = 85%–96%). Of the 164 persons vaccinated ≥20 years previously, the estimated seropositivity rate was 80% (CI = 74%–86%).

## Yellow Fever Vaccine Booster Dose Safety Data

Serious adverse events, yellow fever vaccine–associated viscerotropic disease (a severe illness similar to wild-type disease), and yellow fever vaccine-associated neurologic disease were considered critical risks to assess the need for yellow fever vaccine booster doses (7).

### Serious adverse events

Nine observational studies provided data on serious adverse events for 333 million distributed doses of yellow fever vaccine (3). Overall, 1,255 persons were reported to have a serious adverse event after yellow fever vaccination. For most (84%) persons, it was unknown if the adverse event occurred after a primary or booster dose of the vaccine. Of the 201 persons with a serious adverse event where dose type was known, 14 (7%) of the adverse events occurred after a booster dose of vaccine.

### Viscerotropic disease

Eight observational studies provided data on viscerotropic disease for 437 million distributed doses of yellow fever vaccine (3). A total of 72 persons had yellow fever vaccine–associated viscerotropic disease. Of the 31 persons where dose type was known, one (3%) had viscerotropic disease after receiving a booster dose of the vaccine; no laboratory testing to assess vaccine causality was performed for that case.

BOX Recommendations for use of yellow fever vaccine booster doses*

**Table:** 

A single primary dose of yellow fever vaccine provides long-lasting protection and is adequate for most travelers [Category A]. Additional doses of yellow fever vaccine are recommended for certain travelers: – Women who were pregnant (regardless of trimester) when they received their initial dose of yellow fever vaccine should receive 1 additional dose of yellow fever vaccine before their next travel that puts them at risk for yellow fever virus infection [Category A]; – Persons who received a hematopoietic stem cell transplant after receiving a dose of yellow fever vaccine and who are sufficiently immunocompetent to be safely vaccinated should be revaccinated before their next travel that puts them at risk for yellow fever virus infection [Category A]; – Persons who were infected with human immunodeficiency virus when they received their last dose of yellow fever vaccine should receive a dose every 10 years if they continue to be at risk for yellow fever virus infection [Category A]. A booster dose may be given to travelers who received their last dose of yellow fever vaccine at least 10 years previously and who will be in a higher-risk setting based on season, location, activities, and duration of their travel [Category B]. This would include travelers who plan to spend a prolonged period in endemic areas or those traveling to highly endemic areas such as rural West Africa during peak transmission season or an area with an ongoing outbreak. Laboratory workers who routinely handle wild-type yellow fever virus should have yellow fever virus–specific neutralizing antibody titers measured at least every 10 years to determine if they should receive additional doses of the vaccine. For laboratory workers who are unable to have neutralizing antibody titers measured, yellow fever vaccine should be given every 10 years as long as they remain at risk [Category A].

### Neurologic disease

Eight observational studies provided neurologic disease data for approximately 462 million distributed doses of yellow fever vaccine (3). A total of 218 persons had yellow fever vaccine–associated neurologic disease. Of the 110 persons where dose type was known, three (3%) persons reported neurologic disease after receiving a booster dose of the vaccine.

## Other relevant evidence

### Pregnant women

The proportion of women who develop yellow fever virus antibodies is variable and might be related to the trimester in which they received the vaccine. Among pregnant women who received yellow fever vaccine primarily in their third trimester, 39% (32 of 83) had evidence of seroconversion to yellow fever virus at 2–4 weeks post-vaccination, compared with 94% (89 of 95) in the general population (12). Of 433 women vaccinated primarily in the first trimester (mean gestational age = 5.7 weeks; CI = 5.2–6.2), 425 (98%) developed yellow fever virus–specific neutralizing antibodies at 6 weeks post-vaccination (13).

### Hematopoietic stem cell transplant recipients

Data are limited on safety and immunogenicity for yellow fever vaccine in hematopoietic stem cell transplant recipients. However, data suggest most recipients become seronegative to live viral vaccine antigens after transplantation (14). Infectious Diseases Society of America guidelines recommend re-administering live viral vaccines, such as measles, mumps, and rubella vaccine and varicella vaccine, to post-transplant patients if the recipient is seronegative and is no longer immunosuppressed (15).

### HIV-infected persons

Two published studies provide immunogenicity data for yellow fever vaccines in HIV-infected persons (16,17). Both studies found lower rates of yellow fever virus–specific neutralizing antibodies among HIV-infected persons compared with uninfected controls at 10 to 12 months post-vaccination. Although the mechanisms for the diminished immune response in HIV-infected persons are uncertain, an inverse correlation exists between immune response and HIV RNA levels and a positive correlation with CD4+ cell counts (18).

### Young children

Twelve studies provided data on the initial immune response to yellow fever vaccine in children aged 4 months–10 years (3). All studies included children who resided in endemic areas, and 10 studies included children who received at least one other vaccine at the same time as yellow fever vaccine. Based on a random effects model, the estimated seroconversion rate in 4,675 children was 93% (CI = 88%–96%). No difference was observed in the seroconversion rates between children aged <9 months and those aged ≥9 months (3).

### Other higher-risk groups

Over the preceding 20 years, 90% of all yellow fever cases were reported from countries in West Africa, and epidemiologic data suggest that travelers to West Africa are at the highest risk for travel-associated yellow fever (5). Persons traveling to an area with an ongoing outbreak, persons traveling for a prolonged period in an endemic area, and laboratory workers who routinely handle wild-type yellow fever virus are also considered to be at higher risk for yellow fever virus exposure and disease than other persons for whom yellow fever vaccine is recommended.

**Summary**

What is currently recommended?

In 2009, the Advisory Committee on Immunization Practices (ACIP) approved yellow fever vaccine recommendations that noted International Health Regulations require revaccination at intervals of 10 years to boost antibody titer. Evidence from multiple studies demonstrates that yellow fever vaccine immunity persists for many decades and might provide life-long protection.

Why are the recommendations being modified now?

The World Health Organization Strategic Advisory Group of Experts in Immunization concluded in April 2013 that a single primary dose of yellow fever vaccine is sufficient to confer sustained immunity and lifelong protection against yellow fever disease, and a booster dose of the vaccine is not needed. In May 2014, the World Health Assembly adopted the recommendation to remove the 10-year booster dose requirement from the International Health Regulations by June 2016. Once the International Health Regulations are updated, the current statement in the ACIP recommendation will no longer be relevant.

What are the new recommendations?

A single primary dose of yellow fever vaccine provides long-lasting protection and is adequate for most travelers. The recommendations also provide considerations and recommendations for at-risk laboratory personnel and certain travelers to receive additional doses of yellow fever vaccine.

## Rationale for Yellow Fever Vaccine Booster Dose Recommendations

The GRADE evaluation found that there are few vaccine failures documented after a primary dose of yellow fever vaccine, most (92%) primary vaccine recipients maintain detectable levels of neutralizing antibodies ≥10 years post-vaccination, and few serious adverse events have been reported after a booster dose of yellow fever vaccine (3). Based on the available data, ACIP voted to no longer recommend booster dose of yellow fever vaccine for most travelers, because a single dose of yellow fever vaccine provides long-lasting protection (Box). However, additional doses of yellow fever vaccine are recommended for certain populations (i.e., pregnant women, hematopoietic stem cell transplant recipients, and HIV-infected persons) who might not have as robust or sustained immune response to yellow fever vaccine compared with other recipients. Furthermore, additional doses may be given to certain groups believed to be at increased risk for yellow fever disease either because of their location and duration of travel or because of more consistent exposure to virulent virus (i.e., laboratory workers). ACIP meeting minutes are available at http://www.cdc.gov/vaccines/acip/meetings/meetings-info.html.

## Footnotes

* Persons being considered for additional doses of yellow fever vaccine should be assessed for contraindications or precautions in accordance with the current yellow fever vaccine ACIP recommendations (7).
